# Intraoperative Colloid Use on Post-operative Renal Function

**DOI:** 10.1007/s40140-024-00607-2

**Published:** 2024-02-23

**Authors:** Jacqueline Chen, Zhengmin Ma, Ke Peng, Fuhai Ji, Nicole Keiko Shirakawa

**Affiliations:** 1 Department of Anesthesiology and Pain Medicine, University of California Davis Health, 4150 V Street, Suite, Sacramento, CA 1200, USA; 2 Department of Anesthesiology, The First Affiliated Hospital of Soochow University, Suzhou, China

**Keywords:** Intraoperative colloids, Acute kidney injury, Albumin, Starches

## Abstract

**Purpose of Review:**

This review summarizes the most recent literature on the association between intraoperative colloid administration and its effect on post-operative renal function.

**Recent Findings:**

It appears albumin decreases the incidence of acute kidney injury (AKI); however, meta-analysis studies show an increased need for post-operative renal replacement therapy. There was moderate certainty that early starches increased the need for renal replacement therapy; however, it appears newer starches have a better safety profile. Gelatins do not appear to contribute to renal dysfunction, despite a study showing a statistically insignificant higher incidence of moderate AKI. Studies involving dextran suggest better renal outcomes when used as a priming solution for cardiopulmonary bypass.

**Summary:**

Albumin administration remains controversial with conflicting studies. While earlier starch products have been associated with renal dysfunction, further studies should be done on newer starches. There are limited studies for gelatins and dextran, suggesting possible renal-sparing effects.

## Introduction

**Fig. 1:**
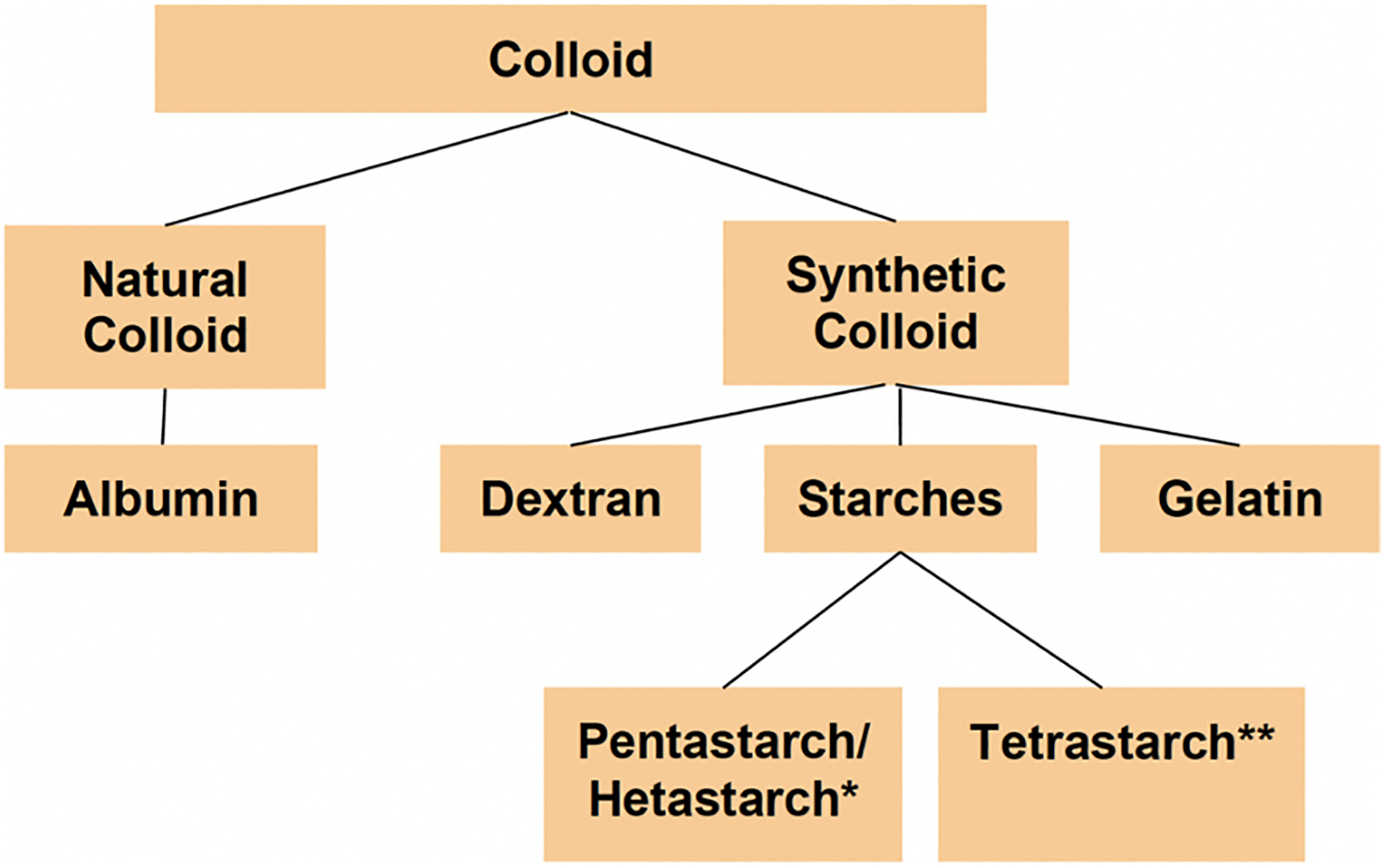
Categorizing the different colloids. Asterisk (*) denotes first-/second-generation starch. Asterisk (**) denotes third-generation starch

Post-operative acute kidney injury (AKI) remains a relatively common perioperative complication in both cardiac and non-cardiac surgery, which has been shown to worsen both short- and long-term outcomes such as progression to chronic kidney disease, cardiovascular complications, and even mortality [1, 2••]. A report from the joint consensus Acute Disease Quality Initiative & Perioperative Quality Initiative states the etiology of perioperative AKI is likely multifactorial, such as hypovolemia, oxidative stress, inflammatory mediators, and nephrotoxins [1]. Of note, perioperative fluid administration is an often-discussed topic among anesthesiology, with hypovolemia-induced post-operative AKI associated with 1–7% of new-onset AKI during the perioperative period for non-cardiac surgery [3]. In cardiac surgery, the incidence of post-operative AKI has been noted to have a prevalence as high as 30% [2, 4]. Such complications are associated with increased healthcare cost, length of hospital stay, morbidity, and even mortality [1, 2••]. Perioperative fluid administration usually consists of either crystalloid, colloid, or blood products; however, there have been many studies that have debated the effects and utility of colloid administration as part of fluid resuscitation during the intraoperative period. This literature review aims to bring readers up to date with the current discussion on intraoperative colloid administration and its effect on post-operative renal function. Colloids have a higher molecular weight and, therefore, have higher oncotic pressure. As a result, colloids have been thought to retain more fluid in the intravascular space, therefore requiring less volume administration for intravascular expansion [5]. Of the fluids, colloids are typically grouped into artificial, such as dextran, starches, gelatin, and natural such as albumin (Fig. 1).

## Albumin

Albumin is considered a natural colloid, as it is synthesized in the liver and contributes to approximately 80% of plasma oncotic pressure [6]. Aside from its contribution to plasma oncotic pressure, it also functions to help in transporting, distributing, and metabolizing both endogenous and exogenous molecules such as fatty acids, hormones, proteins, and medication [5, 6]. Albumin is generally administered in two formulations: 25% and 5%. Twenty-five percent albumin leads to higher intravascular expansion compared to all other colloids, while 5% albumin has similar effects on intravascular expansion as starches (but more than dextran and gelatin). The effects of albumin administration are thought to last between 16 and 24 h [5, 6]. As a natural colloid, albumin is associated with less undesirable effects such as anaphylactoid reactions, coagulopathy, and pruritus when compared with synthetic colloids [7]. Of note, FDA-approved indications for albumin use include hypovolemia with or without shock, volume repletion after paracentesis, hypoalbuminemia, post-dialysis hypotension, hypovolemia due to burn injuries, hemolytic disease of the newborn, and priming of the cardiac surgery bypass circuit [8••]. Non-FDA-approved use of albumin includes use during spontaneous bacterial peritonitis, which was shown to decrease renal impairment and improve mortality when administered in conjunction with antibiotics compared to antibiotics alone [8••]. Contraindications to albumin include clinical situations with volume overload, hypersensitivity to any components within albumin, and usage with sterile water (as the combination of sterile water and albumin can lead to hemolysis and AKI) [8••]. Despite the various indications for albumin administration (particularly for hypovolemia and hypotension), it is important to note that the cost of albumin is approximately 60 times more expensive than crystalloid, as it is about $0.50 to $1.00 per mL compared to $0.01 to $0.10 per mL for crystalloids [8••].

## Artificial Colloids

### Dextran

Dextrans are an artificial colloid synthesized by a bacterial enzyme called dextran sucrase, which could be found in the bacteria *Leuconostoc mesenteroides* [9]. It is a highly branched polysaccharide molecule and has a greater intravascular volume expansion compared to albumin and starches [6]. However, despite the greater effects of intravascular volume expansion, dextran solutions are associated with acute renal failure, likely due to its accumulation in the renal tubules. Other associated negative side effects include more severe anaphylaxis when compared to gelatins or starch solutions and coagulopathy due to its effect on platelet adhesion, fibrinolysis, and Factor VIII. Dextran solutions are primarily excreted by the kidneys and are thought to remain in intravascular plasma for 6 to 12 h [6, 9]. The two most widely used dextran solutions are the 6% and 10% (also referred to as dextran 40 and dextran 70, respectively) [9].

### Gelatins

Gelatins are a collagen-based colloid made from boiling water with animal connective tissue. Since it is rapidly excreted by the kidneys, gelatins have a shorter peak plasma half-life (2.5 h) and shorter duration of action when compared to albumin and starch colloids [5, 6]. As gelatins are relatively smaller molecules when compared to starches, there is less concern for renal impairment and gelatins do not have a threshold for upper limit of transfusions [7]. The benefits of gelatins include cost-effectiveness, ability to transfuse large volumes, and lower likelihood of renal injury. However, gelatins have a higher incidence of anaphylactoid reactions when compared to natural colloid, and there is limited data on the effects of gelatin on coagulopathy [6, 7]. There are primarily three types of gelatin colloid solutions in use today: urea-crosslinked, oxypolygelatins, and succinylated gelatins. In particular, urea-crosslinked gelatins (also called polygelene) contain both calcium (6.5 mmol/L) and potassium (5.1 mmol/L) ions in its solution, making polygelene a beneficial choice to hypocalcemic and/or hypokalemic patients.

### Starches

Hydroxyethyl starches (HES) are a glycogen-resembling synthetic colloid which is made with amylopectin [5, 6, 10]. There are a variety of different HES preparations, distinguished by molecular weight, concentration, and molar substitution. Each of these characteristics impacts the pharmacophysiology upon administration—with concentration affecting the initial volume effect, molecular weight affecting renal excretion, and molar substitution affecting the length of intravascular effect. Starches have a similar intravascular expansion profile as compared to albumin and greater than gelatin; it has an approximate duration of action of 8–12 h [6]. However, despite the cost-effectiveness of starches when compared to albumin, there are significant drawbacks to the first and second-generation starch colloids including coagulopathy, accumulation of colloid in interstitial tissues, and anaphylactoid reactions [6, 11, 12•]. Due to the need to improve the safety of HES, a third-generation HES was developed named tetrastarch. Tetrastarches are noted to have a reduction of transfusion needs and, therefore, are likely not to have the same negative coagulopathic effects when compared to the first- or second-generation starches [12–14•]. Of note, there have also been multiple studies on the effect of both first/second-generation and third-generation HES and the renal system, which will be outlined further down the review.

## Natural Colloid and Renal Function

According to an observational retrospective cohort study, approximately 15% of non-cardiac surgical cases reported administration of iso-oncotic (5%) albumin [15]. Notably, albumin was shown to be more effective as a bolus dose than given in a steady-state infusion rate [16]. In the same study, the authors demonstrated an association between albumin use, AKI, severe AKI, pulmonary complications, and net-positive fluid analysis. However, it is worthy to note that the study endpoints were found to have even more significant associations with large-volume crystalloid administration. Therefore, the study noted that the positive association between post-operative renal complications and albumin administration was likely due to the acuity of the cases (e.g., higher ASA classification, required blood transfusions, continuous vasopressor use, intraoperative hypotension) rather than the colloid administration. In fact, further studies on albumin administration and AKI showed a decreased incidence in post-operative AKI when compared to no albumin administration [17•] In addition, albumin is not an exogenous colloid and has not been found in renal tubules on autopsy [18•].

While it appears there is limited harm in the administration of albumin, there is also limited evidence in support of albumin administration—especially when compared to crystalloids [19•]. When compared with crystalloid administration in cardiac surgery, one recent study showed no significant difference in acute renal failure with albumin administration [19]. In fact, a Cochrane review comprising 22 albumin studies appeared to suggest (with low certainty) that the relative risk for requiring renal replacement therapy was higher with albumin administration in the critically ill patient [20••]. Overall, it appears recent literature concludes a neutral to possibly negative effect of albumin administration on renal function (Table 1).

## Synthetic Colloids and Renal Function

Most recent studies regarding colloid administration and renal function seem to be centered around comparing starches and crystalloid administration. One large retrospective study comparing the incidence of AKI in cardiac surgery after starch administration demonstrated no significant difference when compared to crystalloids [21•]. However, the same Cochrane review from 2018 noted that there was evidence that starch administration increased the need for blood transfusions and renal replacement therapy (with moderate certainty) [20••]. It is important to note, however, that the Cochrane review did not separate the first/second-generation starches from third-generation starches. In particular, there are some newer studies that suggest the third-generation starches are associated with a lower incidence of renal replacement therapy [22•]. Additionally, third-generation starches did not have any higher rates of AKI, worsening of AKI or higher requirements for renal replacement therapy when compared to albumin [21, 22•].

Unfortunately, there are very limited and conflicting studies when it comes to the administration of gelatins and dextrans (Table 1). From the limited studies, it appears that the use of gelatins did not appear to have an association with higher BUN, Cr, urine output, Na, or use of diuretics in liver transplant patients [23•]. Other studies also seem to support the conclusion that the use of gelatins does not have an association with a higher incidence of renal dysfunction [24, 25]. However, there was one study that noted that there was a higher incidence of moderate AKI with gelatin administration in patients receiving cardiac surgery; however, the conclusion of that study stated that the difference was likely insignificant [26]. Overall, it seems as though there is no definitive consensus about both the benefits and risks of gelatin administration.

Recent studies pertaining to dextran administration and renal function appear to mostly be for cardiac surgery and cardiopulmonary bypass—and largely focused on as a secondary endpoint. In a pilot study comparing a dextran-based prime for cardiopulmonary bypass and a crystalloid-based prime, there was found to be no significant difference in creatinine between the two groups [27•] suggesting there is limited evidence of nephrotoxicity with dextran administration. In fact, a follow-up secondary analysis study shows that there is less renal tubular injury when cardiopulmonary bypass is primed with dextran rather than crystalloid [28], suggesting there may in fact be some renal benefit in dextran administration. Of note, there are no recent studies studying the association between dextran use and renal replacement therapy [20••].

## Conclusion

The results from published studies suggested that there are no differences on renal function between the use of albumin and other synthetic colloids and crystalloids and the consensus among the risks and benefits of colloid administration overall is still debated. Given that albumin is anywhere from 5 to 100 times the cost of crystalloid, it is important to weigh the financial cost against the seemingly neutral to slightly negative benefits of administration. While there have been numerous studies on starches and renal function that suggest starch administration has a negative effect on renal function, it is worthwhile to note that many of those studies did not separate the use of first/second-generation starches from the newer third-generation starches. Newer studies focusing on third-generation starches seem to show similar effects on renal function as albumin. Given the cheaper costs of tetrastarch when compared to albumin, it may be beneficial to focus on a non-inferiority study comparing third-generation starch (tetrastarch) and albumin.

## Figures and Tables

**Table 1:** The effect of different colloids on post-operative AKI

Colloid	Study	Design	Treatment	Major findings
Albumin	Pesonen 2022 [18•] Cardiac surgery	Randomized double-blind single-center clinical trial (*n* = 1407)	Albumin vs. Ringer’s acetate	No significant difference in renal failure with albumin administration (*P* = 0.2)
	Lewis 2018 [20••] Critically Ill patients (surgical and non-surgical)	Meta-analysis of randomized control trials control trials (*n* = 3028)	Albumin vs. Crystalloid	Albumin had minimal difference to the need for RRT (RR 1.11, 95% CI 0.96 to 1.27; very low-certainty evidence)
	Kammerer 2018 Urologic surgery	Randomized control single-blind prospective study (*n* = 100)	Albumin vs. Starch	No significant difference in the ratio of serum cystatin C (*P* = 0.165)
	Maleki 2016 Cardiac surgery	Randomized double-blind single-center study (*n* = 60)	Albumin vs. Starch	The serum Cr and GFR at 24, 48 and 72 hours post operation was not significant difference between albumin and starch
Starch	Lewis 2018 [20••] Critically Ill patients (surgical and non- surgical)	Meta-analysis of randomized control trials control trials (*n* = 3028)	Starch vs. Crystalloid	Starches likely increase the need for RRT (RR 1.30, 95% CI 1.14 to 1.48; moderate certainty)
	Miyao 2020 [16] Varieties of surgery	Retrospective propensity-matched cohort study *(n* = 58,425)	Starch vs. Albumin	Third-generation starches have lower incidence of renal replacement therapy (15.2% vs 20.8%). No significant difference in AKI (*P* = 0.08)
	Tobey 2017 [28] Cardiac surgery	Retrospective cohort study (*n* = 1265)	Starch vs. no Starch	The propensity-weighted adjusted OR showed no difference in AKI development between the colloid and noncolloid groups (*P* = 0.775)
	Duncan 2020 Cardiac surgery	Randomized single-center triple-blinded non-inferiority study (*n* = 141)	Starch vs. Albumin	Post-operative urine neutrophil gelatinase-associated lipocalin was slightly lower with starch vs. albumin (*P* = 0.15)
	Miyao 2020 Varieties of surgery	Retrospectively propensity score-matched study (*n* = 578)	Starch vs. Albumin	No statistically significant difference in the incidence of AKI between the starch and the albumin (*P* = 0.08).
Gelatin	Demir 2015 [22•] Liver transplantation	Single-center simple blinded randomized control trial (n=36)	Gelatin vs. Starch	Post-operative Cr higher in Gelatin vs starch (*P*<0.001 vs *P*=0.42). Post-operative eGFR was lower in both Gelatin & starch group (*P*<0.001, *P*=0.004)
	Mohanen 2019 [21•] Abdominal surgery	Single-center prospective randomized control trial (*n* = 66) pilot	Gelatin vs. Starch	Neither gelatin or starch were associated with renal dysfunction (*P* = 0.586)
	Tehran 2022 [23•] Liver transplantation	Single-center randomized control trial (*n* = 140)	Gelatin vs. albumin	No significant difference in renal outcomes (*P* = 0.485)
	Koponen 2022 [24] Cardiac surgery	Prospective cohort study (*n* = 1187)	Gelatin vs. Crystalloid	No significant difference in AKI, Cr or need for renal replacement therapy (*P* = 0.414, 0.689, 0.999)
Dextran	Barbu 2020 [25] Cardiac surgery	Randomized control double-blind prospective study (*n* = 80)	Dextran vs. Crystalloid	No significant difference in Cr (*P* = 0.35)
	Kolsrud 2022 [26] Cardiac surgery	Prospective single-center double-blinded randomized control trial (*n* = 84)	Dextran vs. Crystalloid	Dextran 40-based priming solution led to lower rise of NAG (*P* = 0.038), but no significant difference in Cr or AKI (*P* = 0.76, *P* = 0.66)

*RRT* renal replacement therapy, *RR* risk ratio, *CI* confidence interval, *Cr* creatinine, *GFR* glomerular filtration rate, *AKI* acute kidney injury, *CKD* chronic kidney disease, *eGFR* estimated glomerular filtration rate, *NAG* N-acetyl-β-d-glycosaminidase (NAG)

## References

[1] Prowle JR, Forni LG, Bell M, Chew MS, Edwards M, Grams ME, Grocott MPW, Liu KD, McIlroy D, Murray PT, Ostermann M, Zarbock A, Bagshaw SM, Bartz R, Bell S, Bihorac A, Gan TJ, Hobson CE, Joannidis M, Postoperative acute kidney injury in adult non-cardiac surgery: joint consensus report of the Acute Disease Quality Initiative and PeriOperative Quality Initiative. Nat Rev Nephrol. 2021;17(9):605–18. 10.1038/s41581-021-00418-2. 33976395 PMC8367817

[2.••] Peng K, McIlroy DR, Bollen BA, Billings FT 4th, Zarbock A, Popescu WM, Fox AA, Shore-Lesserson L, Zhou S, Geube MA, Ji F, Bhatia M, Schwann NM, Shaw AD, Liu H. Society of cardiovascular anesthesiologists clinical practice update for management of acute kidney injury associated with cardiac surgery. Anesth Analg. 2022;135(4):744–56. 35544772 10.1213/ANE.0000000000006068

[3] Kashy BK, Podolyak A, Makarova N, Dalton JE, Sessler DI, Kurz A. Effect of hydroxyethyl starch on postoperative kidney function in patients having noncardiac surgery. Anesthesiology. 2014;121:730–9. 10.1097/ALN.0000000000000375. 25054470 PMC4389778

[4] Vives M, Hernandez A, Parramon F, Estanyol N, Pardina B, Muñoz A, Alvarez P, Hernandez C. Acute kidney injury after cardiac surgery: prevalence, impact and management challenges. Int J Nephrol Renovasc Dis. 2019;2(12):153–66. 10.2147/IJNRD.S167477. PMC6612286 31303781

[5] Dubois MJ, Vincent JL. Colloid fluids. In: Hahn RG, Prough DS, Svensen CH, editors. Perioperative fluid therapy. 1st ed. New York: Wiley; 2007. p. 153–611.

[6] Martino P The ICU Book. 3rd ed. Philadelphia: Churchill Livingstone; 2007. Colloid and crystalloid resuscitation. p. 233–54.

[7] Barron ME, Wilkes NRJ. A systematic review of the comparative safety of colloids. Arch Surg. 2004;139:552–63. 15136357 10.1001/archsurg.139.5.552

[8.••] Campos Munoz A, Jain NK, Gupta M. Albumin colloid. [Updated 2022 Jun 27]. In: StatPearls [Internet]. Treasure Island (FL). StatPearls Publishing; 2023. Available from: https://www.ncbi.nlm.nih.gov/books/NBK534241/ . Accessed 15 Jan 2023.

[9] Mitra S, Khandelwal P. Are all colloids same? How to select the right colloid? Indian J Anaesth. 2009;53(5):592–607. 20640110 PMC2900092

[10] Westphal M, MFM J, Kozek-Langenecker SA, Stocker R, Guidet B, Van Aken H. Hydroxyethyl starches. Different products—different effects. Anesthesiology. 2009;111:187–202. 19512862 10.1097/ALN.0b013e3181a7ec82

[11] Schortgen F, Effects of hydroxyethylstarch and gelatin on renal function in severe sepsis: a multicentre randomized study. Lancet. 2001;357:911–6. 11289347 10.1016/S0140-6736(00)04211-2

[12.•] Davidson IJ. Renal impact of fluid management with colloids: a comparative review. Eur J Anaesthesiol. 2006;23:721–38. 16723059 10.1017/S0265021506000639

[13] Kozek-Langenecker SA, Jungheinrich C, Sauermann W, van der Linden PJ. The effects of hydroxyethyl starch 130/0.4 (6%) on blood loss and use of blood products in major surgery: a pooled analysis of randomized clinical trials. Anesth Analg. 2008;107:382–90. 18633012 10.1213/ane.0b013e31817e6eac

[14.•] Sakr Y, Payen D, Reinhart K, Sipmann F, Zavala E, Bewley J, Effects of hydroxyethyl starch administration on renal function in critically ill patients. Br J Anaesth. 2007;98:216–224. 17251213 10.1093/bja/ael333

[15] Lazzareschi DV, Fong N, Mavrothalassitis O, Whitlock EL, Chen CL, Chiu C, Adelmann D, Bokoch MP, Chen LL, Liu KD, Pirracchio R, Romain M, Michael R, Legrand M, for the MPOG Collaborators. Intraoperative use of albumin in major non-cardiac surgery: incidence, variability, and association with outcomes. Ann Surg. 2022; 10.1097/SLA.0000000000005774. PMC10481928 36521076

[16] Statkevicius S, Bonnevier J, Fisher J, Bark BP, Larsson E, Öberg CM, Kannisto P, Tingstedt B, Bentzer P. Albumin infusion rate and plasma volume expansion: a randomized clinical trial in postoperative patients after major surgery. Crit Care. 2019;23(1):191. 10.1186/s13054-019-2477-7. 31138247 PMC6537197

[17.•] Xiang F, Huang FH, Huang JP, Li X, Dong NG, Xiao YB, Zhao Q, Xiao LQ, Zhang HT, Zhang C, Cheng ZY, Chen LW, Chen JM, Wang HS, Guo YQ, Liu N, Luo Z, Hou XT, Ji BY, Expert Consensus on the Use of Human Serum Albumin in Adult Cardiac Surgery. Chin Med J. 2023;XX:1–9. 10.1097/CM9.0000000000002709. PMC10278724 37083122

[18.•] Hryciw N, Joannidis M, Hiremath S, Callum J, Clark EG. Intravenous albumin for mitigating hypotension and augmenting ultrafiltration during kidney replacement therapy. Clin J Am Soc Nephrol. 2021;16(5):820–8. 10.2215/CJN.09670620. 33115729 PMC8259476

[19.•] Pesonen E, Vlasov H, Suojaranta R, Hiippala S, Schramko A, Wilkman E, Eränen T, Arvonen K, Mazanikov M, Salminen US, Meinberg M, Vähäsilta T, Petäjä L, Raivio P, Juvonen T, Pettilä V. Effect of 4% albumin solution vs ringer acetate on major adverse events in patients undergoing cardiac surgery with cardiopulmonary bypass: a randomized clinical trial. JAMA. 2022;328(3):251–8. 10.1001/jama.2022.10461. 35852528 PMC9297113

[20.••] Lewis SR, Pritchard MW, Evans DJW, Butler AR, Alderson P, Smith AF, Roberts I. Colloids versus crystalloids for fluid resuscitation in critically ill people. Cochrane Database of Systematic Reviews. 2018;(8):CD000567. 10.1002/14651858.CD000567.pub7. PMC6513027 30073665

[21.•] Tobey R, Cheng H, Gao M, Li Z, Young N, Boyd D, Ji F, Liu H. Postoperative AKI and blood product transfusion after synthetic colloid use during cardiac surgery. J Cardiothorac Vasc Anesth. 2017;31(3):853–62. 28302346 10.1053/j.jvca.2016.12.024 PMC5489358

[22.•] Miyao H, Kotake Y. Postoperative renal morbidity and mortality after volume replacement with hydroxyethyl starch 130/0.4 or albumin during surgery: a propensity score-matched study. J Anesth. 2020;34(6):881–91. 10.1007/s00540-020-02838-z. 32783070 PMC7674565

[23.•] Tehran SG, Khosravi MB, Sahmeddini MA, Comparing the effect of administering gelatin-low dose albumin versus albumin on renal function in liver transplantation: a randomized clinical trial. Clin Transplant. 2022;36:e14791. 10.1111/ctr.14791. 35950553

[24] Mohanan M, Rajan S, Kesavan R, Mohamed ZU, Ramaiyar SK, Kumar L. Evaluation of renal function with administration of 6% hydroxyethyl starch and 4% gelatin in major abdominal surgeries: a pilot study. Anesth Essays Res. 2019;13(2):219–24. 31198234 10.4103/aer.AER_25_19 PMC6545968

[25] Demir A, Aydınlı B, Toprak HI, Impact of 6% starch 130/0.4 and 4% gelatin infusion on kidney function in living-donor liver transplantation. Transplant Proc. 2015;47(6):1883–9. 26293067 10.1016/j.transproceed.2015.05.015

[26] Koponen T, Musialowicz T, Lahtinen P. Gelatin and the risk of acute kidney injury after cardiac surgery. Acta Anaesthesiol Scand. 2022;66(2):215–22. 10.1111/aas.14004. 34811729

[27.•] Barbu M, Kolsrud O, Ricksten SE, Dellgren G, Zetterberg H, Blennow K, Björk K, Thorén A, Hansson C, Jeppsson A. Dextran- versus crystalloid-based prime in cardiac surgery: a prospective randomized pilot study. Ann Thorac Surg. 2020;110(5):1541–7. 10.1016/j.athoracsur.2020.03.031. 32302659

[28] Kolsrud O, Barbu M, Dellgren G, Björk K, Corderfeldt A, Thoren A, Jeppsson A, Ricksten SE. Dextran-based priming solution during cardiopulmonary bypass attenuates renal tubular injury-a secondary analysis of randomized controlled trial in adult cardiac surgery patients. Acta Anaesthesiol Scand. 2022;66(1):40–7. 10.1111/aas.13975. 34424995

